# Antiproliferative Activity of Non-Calcemic Vitamin D Analogs on Human Melanoma Lines in Relation to VDR and PDIA3 Receptors

**DOI:** 10.3390/ijms19092583

**Published:** 2018-08-31

**Authors:** Tomasz Wasiewicz, Anna Piotrowska, Justyna Wierzbicka, Andrzej T. Slominski, Michal A. Zmijewski

**Affiliations:** 1 Department of Histology, Medical University of Gdansk, 80-210 Gdansk, Poland; tomwasxp@poczta.onet.pl (T.W.); annapiotrowska@gumed.edu.pl (A.P.); justyna.wierzbicka@gumed.edu.pl (J.W.); 2 Department of Dermatology, University of Alabama, Birmingham, AL 35294, USA; aslominski@uabmc.edu; 3 Comprehensive Cancer Center, Cancer Chemoprevention Program, University of Alabama, Birmingham, AL 35294, USA; 4 VA Medical Center, Birmingham, AL 35294, USA

**Keywords:** vitamin D, 1,25(OH)_2_D_3_, calcipotriol, 21(OH)pD, vitamin D analogs, melanoma, human melanoma cell lines, VDR translocation, anti-melanoma activity

## Abstract

Vitamin D is a precursor for secosteroidal hormones, which demonstrate pleiotropic biological activities, including the regulation of growth and the differentiation of normal and malignant cells. Our previous studies have indicated that the inhibition of melanoma proliferation by a short side-chain, low calcemic analog of vitamin D—21(OH)pD is not fully dependent on the expression of vitamin D receptor (VDR). We have examined the effects of classic vitamin D metabolites, 1,25(OH)_2_D_3_ and 25(OH)D_3_, and two low calcemic vitamin D analogs, (21(OH)pD and calcipotriol), on proliferation, mRNA expression and vitamin D receptor (VDR) translocation in three human melanoma cell lines: WM98, A375 and SK-MEL-188b (subline b of SK-MEL-188, which lost responsiveness to 1,25(OH)_2_D_3_ and became VDR^−/−^CYP27B1^−/−^). All tested compounds efficiently inhibited the proliferation of WM98 and A375 melanoma cells except SK-MEL-188b, in which only the short side-chain vitamin D analog—21(OH)pD was effective. Overall, 21(OH)pD was the most potent compound in all three melanoma cell lines in the study. The lack of responsiveness of SK-MEL-188b to 1,25(OH)_2_D_3_, 25(OH)D_3_ and calcipotriol is explained by a lack of characteristic transcripts for the *VDR*, its splicing variants as well as for vitamin D-activating enzyme CYP27B1. On the other hand, the expression of *VDR* and its splicing variants and other vitamin D related genes (*RXR*, *PDIA3*, *CYP3A4*, *CYP2R1*, *CYP27B1*, *CYP24A1* and *CYP11A1*) was detected in WM98 and A375 melanomas with the transcript levels being modulated by vitamin D analogs. The expression of *VDR* isoforms in WM98 cells was stimulated strongly by calcipotriol. The antiproliferative activities of 21(OH)pD appear not to require VDR translocation to the nucleus, which explains the high efficacy of this noncalcemic pregnacalciferol analog in SK-MEL-188b melanoma, that is, VDR^−/−^. Therefore, we propose that 21(OH)pD is a good candidate for melanoma therapy, although the mechanism of its action remains to be defined.

## 1. Introduction

Vitamin D is a naturally occurring hormone precursor, which after activation serves as a pleiotropic regulator of homeostasis on cellular and whole organism levels [1,2]. It is produced in the skin which is subjected to the ultraviolet B (UVB) fraction of sunlight or could be acquired from nutritional sources and supplementation. UVB exposure results in the conversion of epidermal 7-dehydrocholesterol (7DHC) into pre-vitamin D_3_, which undergoes subsequent isomerization to vitamin D_3_ [2,3,4,5]. Vitamin D_3_ has to be hydroxylated in order to implement its biological activities. The first step takes place in the liver, where vitamin D_3_ is converted to 25(OH)D_3_ (25-hydroxyvitamin D_3_, calcifediol) by CYP2R1, CYP27A1 or CYP3A4 (CYP3A4 belongs to cytochrome P450 superfamily and is involved in drug metabolism, but also has a capacity for vitamin D hydroxylation), and 25(OH)D_3_ is subsequently hydroxylated by 1α-hydroxylase (CYP27B1) in the kidney. In addition, 1,25(OH)_2_D_3_ can be generated locally in many cell types (keratinocytes, dendritic cells, melanocytes, lymphocytes and cancer cells) expressing appropriate enzymatic machinery [6,7]. The level of 1,25(OH)_2_D_3_ and its metabolites in circulation are tightly regulated through a negative feedback loop by 24-hydroxylase (CYP24A1), which metabolizes calcitriol into water-soluble and inactive calcitroic acid [8]. The catalytically inactive splicing variant of *CYP24A1* (*CYP24SV*) may serve as dominant negative regulator of vitamin D_3_ catabolism and possibly contribute to the extracellular accumulation of 1,25(OH)2D_3_ [9]. Most recently, it was discovered that CYP11A1, the rate limiting enzyme of steroidogenesis, also metabolizes 7DHC, vitamin D_3_ and lumisterol to their corresponding hydroxyderivatives with full and short side-chains [10,11,12,13,14,15].

According to the genomic pathway, active forms of vitamin D_3_ exert their biological activities through interactions with the VDR, which is expressed by a majority of cells in the body [16,17]. Activated VDR forms a heterodimer with retinoid X receptor (RXR), and this complex is translocated to the nucleus where it binds to the VDREs (vitamin D response elements) and activates the expression of hundreds of human genes [17,18]. The activity of this complex is also regulated by the recruitment of co-activators or co-repressors to modulate the expression of selected genes, including those participating in the inhibition of cell cycle progression and the stimulation of differentiation and apoptosis [19,20,21,22].

In addition to the well-characterized vitamin D_3_ genomic actions, its analogs may also exert a rapid, non-genomic response (RR: rapid response) [23,24,25]. There is growing evidence that the co-localization of VDR [26,27] with caveolae of plasma membrane, as well as the binding of vitamin D to PDIA3 (Protein disulfide-isomerase A3), is responsible for the alternative (non-genomic) action of vitamin D_3_ and its analogs [23,28].

According to epidemiological studies, the incidence of human malignant melanoma has been increasing steadily since the 1970s [29,30,31,32]. Early detection of melanoma results in a good prognosis for patients; however, the survival rate and therapeutic procedures for advanced or metastatic melanomas are very limited. Ultraviolet radiation, in addition to being fully carcinogenic and a main cause of melanoma [32,33,34], is also the most important factor promoting the formation of vitamin D_3_. The anti-melanoma activity of vitamin D derivatives have been reported previously [35,36,37,38,39,40]. In addition, the presence of specific polymorphisms of *VDR* [41,42,43] or a decreased expression of *VDR*, *CYP27B1*, *CYP24A1* and defects in vitamin D signaling are linked to more advanced stages of melanoma or poorer prognosis [44,45,46,47,48]. Furthermore, proper supplementation with vitamin D is believed to be an important factor in cancer prevention [47,49,50,51].

**Figure 1:**
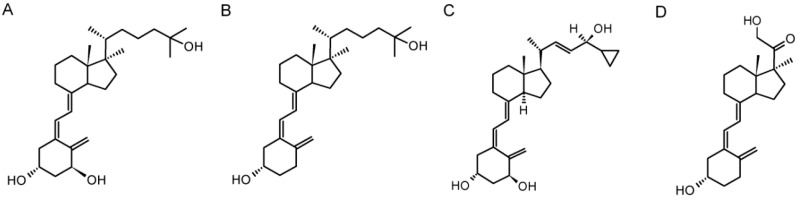
Chemical structure of vitamin D_3_ analogs: 1,25(OH)_2_D_3_ (**A**), 25(OH)D_3_ (**B**), calcipotriol (**C**) and 21(OH)pD (**D**).

Active forms of vitamin D_3_ show antiproliferative properties against several types of cancer, including colorectal cancer [52], breast cancer [53], prostate cancer [54] and melanoma [39,40,55,56,57,58,59]. Furthermore, the use of 1,25(OH)_2_D_3_ as an anticancer drug in very high concentrations (above 50,000 units/day) is currently under clinical investigation; however, it has to be acknowledged that it could potentially cause hypercalcemia [40,50,60]. Since vitamin D_3_ analogs with modified or shortened side-chains were shown to have low or no effect on calcium levels [59,61,62,63], they are potentially better alternatives for calcitriol. So far, more than 3000 vitamin D_3_ analogs have been synthesized, and their biological activity is still intensively investigated as a single agent or in combination with other cytostatics [40,64,65,66]. In this study, we have investigated the response of three human melanoma cell lines against four vitamin D_3_ analogs. We have analyzed the antiproliferative potential of analogs (Figure 1) and found that 21(OH)pD inhibits melanoma growth through a mechanism independent from VDR or PDIA3.

## 2. Results

### 2.1. New Vitamin D_3_ Analogs Effectively Inhibit A375 Cell Proliferation

Our previous studies on human melanomas SK-MEL-188 [10,39,57,59,62,67,68,69], A375 [55], and WM98 [39,68] demonstrated the sensitivity of melanoma cells to vitamin D_3_ analogs with short and full side-chains. The efficacy of compounds was attenuated by melanogenesis, which was associated with the downregulation of VDR expression [56,68]. However, it was also shown that the antiproliferative activity of short side-chain analogs of vitamin D_3_, such as 21(OH)pD, were not fully dependent on VDR expression in rodent melanomas [56], consistent with the poor docking score on the ligand binding domain of the VDR and the poor translocation of VDR to the nucleus [69].

**Figure 2:**
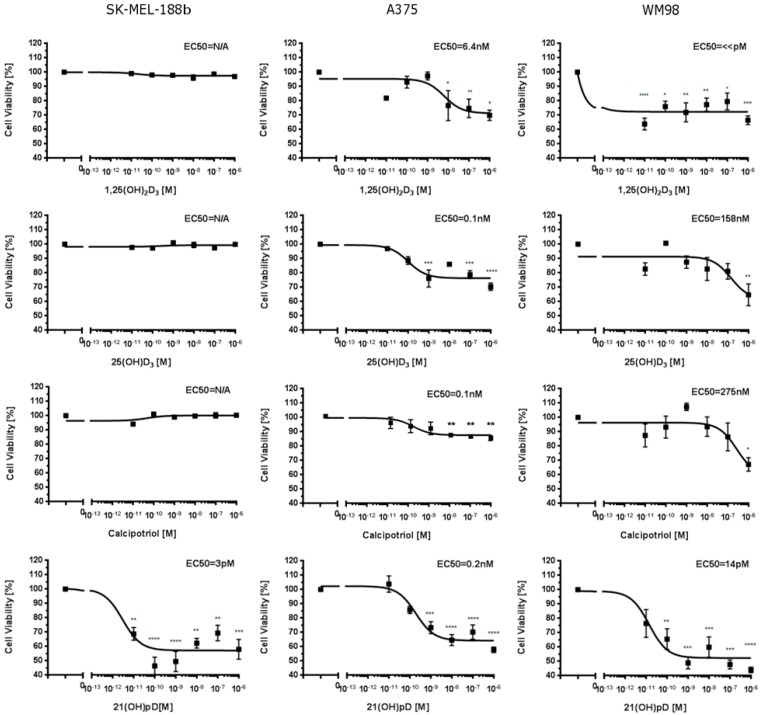
Effects of 1,25(OH)_2_D_3_, 25(OH)D_3_, calcipotriol and 21(OH)pD on growth of human SK-MEL-188b, A375 and WM98 melanoma cells. Cells were seeded into 96-well plates and incubated in a medium supplemented with serial dilution of vitamin D analogs from 0.01 nM to 1000 nM concentration (as described in Material and Methods). The statistical significance of results has been analyzed using one-way ANOVA (GraphPad Software, San Diego, CA, USA) and data are presented as means ± SEM for at least three independent measurements. The cutoff point of significance was defined as *p* < 0.05 (* *p* < 0.05, ** *p* < 0.01, *** *p* < 0.001, **** *p* < 0.0001).

To better understand the effect of VDR expression on the differential action of vitamin D_3_ analogs, we used three human melanoma lines, A375 and WM98 and a subline b of SKMEL-188 which lost responsiveness to vitamin D during in vitro passaging [55], and detected the VDR expression and sensitivity to selected vitamin D analogs (1,25(OH)_2_D_3_, 25(OH)D_3_, 21(OH)pD and calcipotriol). As expected, these compounds [40,65] effectively inhibited the proliferation of A375 and WM98 melanomas expressing VDR receptor. IC_50_ values ranged from pM to µM, and the effects on cell proliferation were strongly dependent on the melanoma cell line and the nature of the compound. Interestingly, non-pigmented SK-MEL-188b was found to be resistant to vitamin D_3_ analogs with a full-length side-chain (1,25(OH)_2_D_3_, 25(OH)D_3_ and calcipotriol) and sensitive to short side-chained 21(OH)pD (Figure 2). Overall, 21(OH)pD was found to be the most potent inhibitor of growth in tested melanoma cell lines.

### 2.2. The Effect of Vitamin D_3_ Compounds on the Expression of Genes Related to Vitamin D_3_ Action or Metabolism

**Figure 3:**
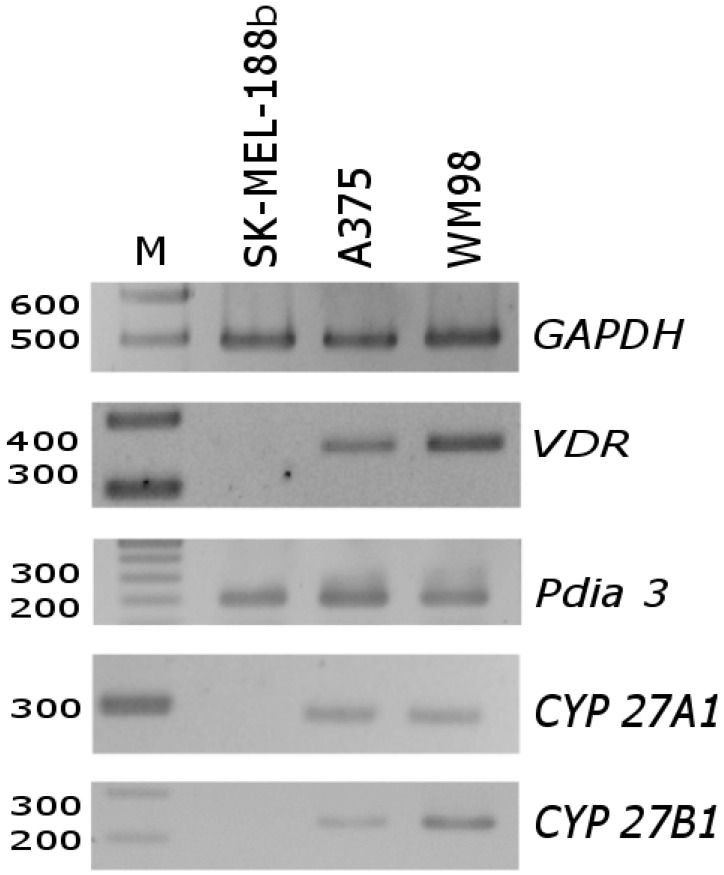
Differences in mRNA level of *VDR*, *PDIA3*, *CYP27A1* and *CYP27B1* genes in SK-MEL-188b cells in comparison with A375 and WM98 cells. Glyceraldehyde 3-phosphate dehydrogenase (GAPDH) was used as a reporter gene to normalize all samples. See Table S1 for primer sequences and the predicted length of PCR fragments.

First, we investigated the expression of vitamin D_3_-related genes in untreated melanoma cells. As shown in Figure 3, PCR fragments characteristic for *VDR*, *CYP27A1* and *CYP27B1* mRNAs were below the level of detectability in SK-MEL-188b melanoma, which did not respond to vitamin D. Only the mRNA of *PDIA3* was detected in this line. On the other hand, in A375 and WM98, cell lines corresponding to transcripts for all tested genes were detected.

**Figure 4:**
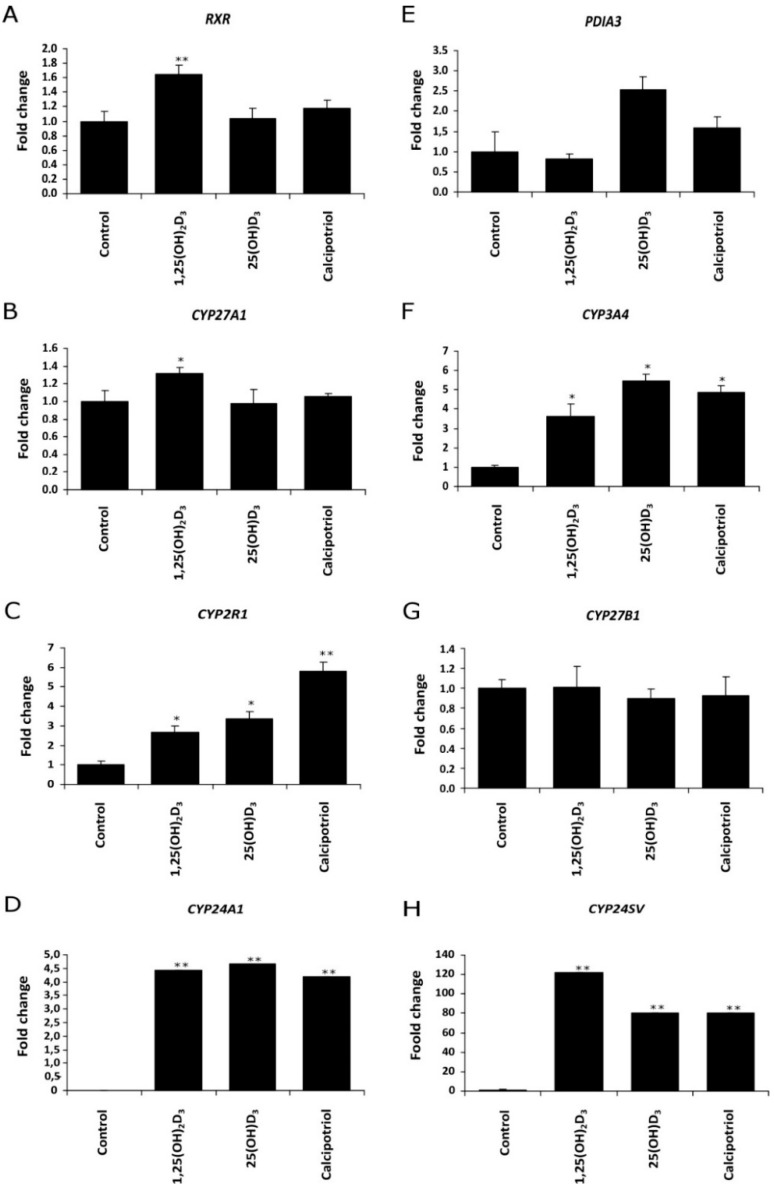
Vitamin D analogs treatment modulate the expression of (**A**) retinoid X receptor (*RXR*), (**E**) disulfide isomerase (*PDIA3*), 25-hydoxylases ((**B**) *CYP27A1*, (**C**) *CYP2R1* or (**F**) *CYP3A4*, (**G**) 1α-hydroxylase (*CYP27B1*)) and 24-hydroxylases ((**D**) *CYP24A1* or (**H**) *CYP24SV*) in WM98 melanoma cell line. Cells were stimulated with 1 µM 1,25(OH)_2_D_3_, 25(OH)D_3_ or calcipotriol for 24 h. Quantitative PCR analyses were performed as described in Materials and Methods. Statistical significance was estimated using *t*-test and data are presented as means ± SD (*n* = 3). The cutoff point for significance is defined as *p* < 0.05 (* *p* < 0.05, ** *p* < 0.01).

Next, we tested the effects of vitamin D_3_ analogues on the relative mRNA levels of vitamin D_3_-related genes in responsive melanoma cell lines (A375 and WM98). The treatment of WM98 melanoma with full length side-chain vitamin D_3_ analogs (1,25(OH)_2_D_3_, 25(OH)D_3_ and calcipotriol) is presented in Figure 4. Only 1,25(OH)_2_D_3_ stimulated the expression of *VDR* Co-receptor *RXR* and of *CYP27A1* (Figure 4B). All three analogs, however, stimulated the expression of *CYP3A4*, *CYP2R1, CYP24A1* and its splicing variant *CYP24A1sv*. Interestingly, the strongest effect (approximately a 6-fold induction) on *CYP2R1* mRNA level was observed in cells treated with calcipotriol (Figure 4C). Furthermore, the level of *CYP27B1* mRNA was not affected by tested secosteroids (Figure 4G), while the expression of *PDIA3* was stimulated only by 25(OH)D_3_ (Figure 4E).

**Figure 5:**
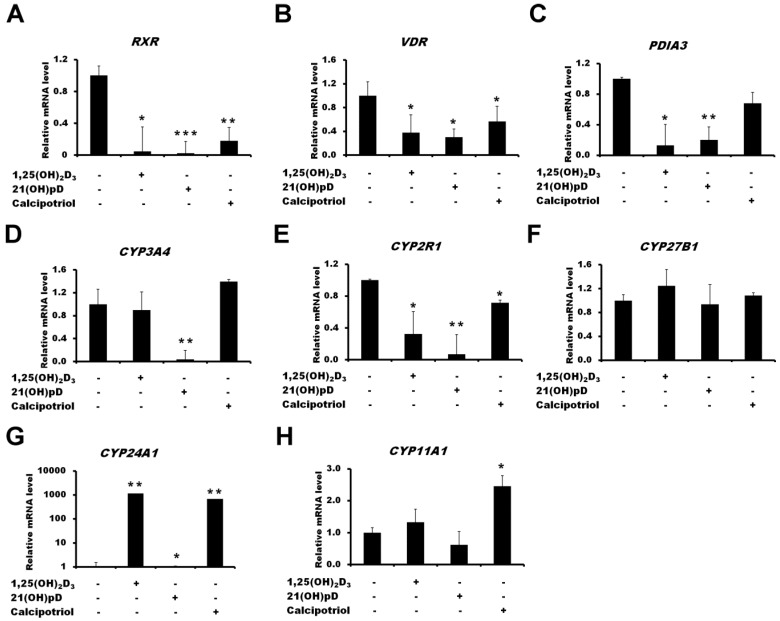
Effects of vitamin D compounds (1,25(OH)_2_D_3_, 21(OH)pD or calcipotriol) on *RXR* (**A**), *VDR* (**B**), *PDIA3* (**C**), *CYP3A4* (**D**), *CYP2R1* (**E**), *CYP27B1* (**F**), *CYP24A1* (**G**) and *CYP11A1* (**H**) genes expression in A375 melanoma cells. A375 melanoma cells were incubated with 100 nM of 1,25(OH)_2_D_3_, 21(OH)pD or calcipotriol for 24 h. mRNA levels were measured by qPCR. Data are shown as means ± S.D of three independent experiments carried out in duplicate. The cutoff point for significance is defined as *p* < 0.05 (* *p* < 0.05, ** *p* < 0.01, *** p < 0.001).

The treatment of A375 melanoma with four vitamin D_3_ analogs (1,25(OH)_2_D_3_, calcipotriol and 21(OH)pD) decreased the expression of *VDR, RXR, PDIA3, CYP2R1* genes (Figure 5). Interestingly, the strongest decrease in the mRNA levels of *CYP3A4* and *CYP2R1* was observed in A375 melanoma cells treated with 21(OH)pD. The induction of *CYP24A1* gene was hundreds of times folds higher in cells treated with 1,25(OH)_2_D_3_ and calcipotriol when compared to 21(OH)pD, showing minimal effect. Finally, the mRNA level of *CYP27B1* was not affected by vitamin D analogues and *CYP11A1* was elevated in A375 melanoma cells only after treatment with calcipotriol.

**Figure 6:**
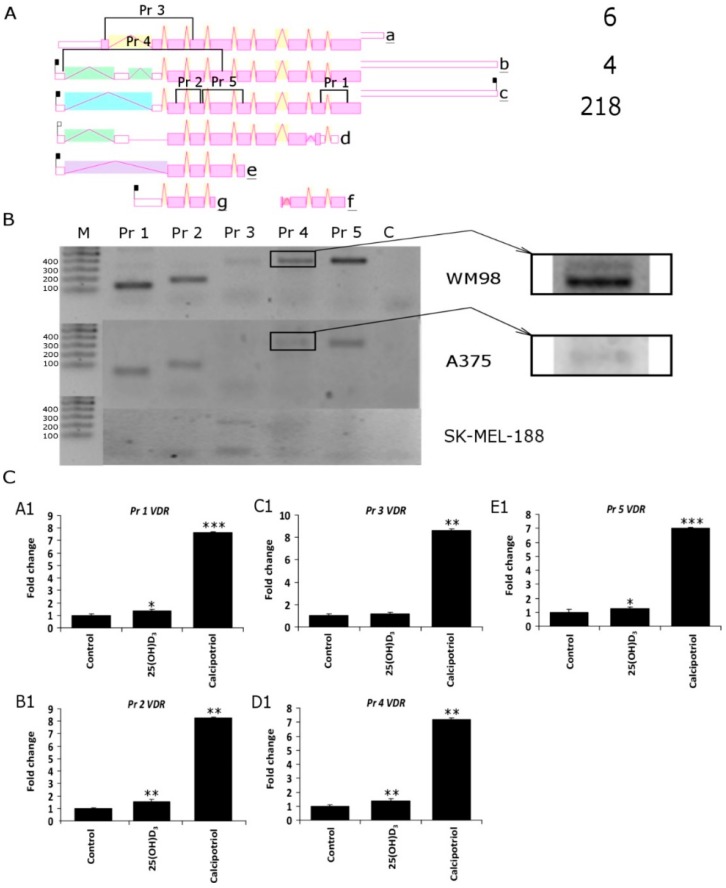
(**A**) Five different PCR primers sets were designed (Pr 1–Pr 5) in order to detect the expression of *VDR* splicing variants in WM98, A375 and SK-MEL-188b cell lines. The most common isoforms are a b and c and the rarely identified isoforms are d, e, f and g. The positions of sets of primers designed to differentiate *VDR* isoforms is also shown. Product 1 (Pr 1) of 132 b.p. is characteristic for isoforms a, b, c and f; Product 2 (Pr 2) of 180 b.p. is universal for all isoforms accept f; Product 3 (Pr 3) of 386 b.p. is unique for isoform a; Product 4 (Pr 4), depending on length of the PCR fragment, indicates isoform “b” 532 b.p. or “c” 410 b.p.; Product 5 (Pr 5) of 384 b.p. is characteristic for isoforms a, b, c, d and e, but not g and f (diagram of VDR splicing variants taken from AceView https://www.ncbi.nlm.nih.gov/IEB/Research/Acembly/). (**B**) Semiquntitative PCR was used to differentiate the *VDR* isoforms (see Material and Methods for details); molecular weight marker (M) as described in. (**C**) Effects of vitamin D analogs on the expression of *VDR* isoforms were analyzed in WM98 melanoma cells. Melanoma cells were treated with 25(OH)D_3_ or calcipotriol at 1 µM concentration for 24 h. Statistical significance was estimated using *t*-test and data are presented as means ± SD (*n* = 3). The cutoff point for significance is defined as *p* < 0.05 (* *p* < 0.05, ** *p* < 0.01, *** *p* < 0.001).

It is well established that the antiproliferative effects of vitamin D_3_ analogs strongly depend on the expression of vitamin D receptor VDR [40,47,65]. As shown in Figure 3, the melanoma cell lines used in the study differ in their basal level of *VDR* expression, and the transcript is absent in SK-MEL-188b, which is not responsive to vitamin D. Theoretically, an alternative splicing of *VDR* pre-mRNA may results in the production of at least five alternative transcripts (Figure 6A); thus, the unique set of primers was designed in order to detect specific variants of *VDR* mRNA (Table S1). Detection of specific variants of *VDR* was confirmed by sequencing of PCR fragments previously separated on agarose gel (Figure 6B). WM98 and A375 melanoma cell lines express all four PCR fragments (Pr 1, Pr 2, Pr 4 and Pr 5) corresponding to *VDR* splicing variant c (Figure 6A). In the WM98 melanoma, the cell line presence of an mRNA characteristic for isoforms a (Pr 3) and b (Pr 4, upper band) was also detected. Interestingly, in SK-MEL-188b melanoma cells, only one fragment of mRNA corresponding to the alternative promoter region of *VDR* (isoform c) was detected. Finally, the effects of two vitamin D_3_ analogs 25(OH)D_3_ and calcipotriol on the alternative splicing of *VDR* pre-mRNA was tested in WM98 cell line (Figure 6C). It was show that treatment with calcipotriol, but not 25(OH)D_3_, resulted in at least 7-fold elevation of mRNA levels corresponding to all fragments of the *VDR* tested (fragments detected by a set of primers: Pr 1 to Pr 5).

### 2.3. Translocation of Vitamin D Receptor by Secosteroids

**Figure 7:**
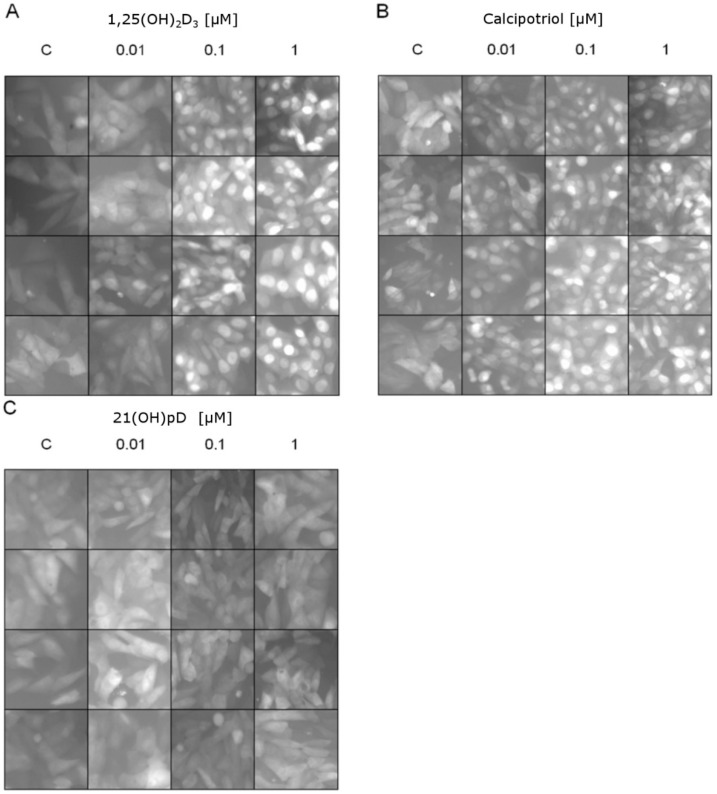
Effects of vitamin D analogs: (**A**) 1,25(OH)_2_D_3_, (**B**) calcipotriol or (**C**) 21(OH)pD_3_ at 0 (C—control), 0.01, 0.1 or 1 μM concentrations on VDR translocation to the nucleus (green fluorescence shown in a gray scale). Melanoma A375 cells were transduced using pLenti-CMV-VDR-GFP-pgkpuro construct: see Materials and Methods. Vitamin D analogs except 21(OH)pD induce VDR translocation to the nucleus after 1 h of incubation in a concentration-dependent manner. Each panel shows four random micrographs for each concentration taken by fluorescent microscope under 100× magnification.

The ligand-induced translocation of VDR receptor into the nucleus was studied using A375 cells stably transduced with pLenti-CMV-VDR-EGFP lentiviral construct [70]. 1,25(OH)_2_D_3_ or calcipotriol induced a dose-dependent and efficient translocation of VDR-GFP fusion protein into the nucleus (Figure 7). A lack of VDR-GFP translocation to the nucleus was observed in cells treated with 21(OH)pD.

## 3. Discussion

There is growing evidence that vitamin D_3_ analogs are excellent candidates for melanoma therapy [40,47,65,71,72]. This is supported by the strong correlation between an adequate level of 25(OH)D_3_ (>30 ng/mL in the serum) and a decreased incidence or severity of several cancers, including melanoma [62,73,74,75,76,77]. However, the use of an active form of vitamin D_3_, 1,25(OH)_2_D_3_ is limited due to its potential hypercalcemic effects; thus, the development and biological evaluation of new low calcemic vitamin D_3_ derivatives is highly desirable [40,55,65,72].

Here, we present results showing the different sensitivities of three melanoma cell lines (SK-MEL-188b, A375 and WM98) to vitamin D_3_ analogs. Recently, we described vitamin D_3_-resistant clone of SK-MEL1-88 (SK-MEL-188b) [55]. Contrary to parental SK-MEL-188, this clone was insensitive to the treatment with 1,25(OH)_2_D_3_, or with several analogs of vitamin D_2_, except of PRI-1731 with the inverse orientation of A ring in comparison to parental 1,25(OH)_2_D_3_ by the introduction of (*5Z*,*7Z*) modification [55,78,79]. This clone does not express the coding region of *VDR* and of *CYP27B1*. Therefore, we call it SK-MEL-188*VDR^−/−^CYP27B1^−/−^*. Interestingly, a short side-chain analog of vitamin D_3_ with a pregnacalciferol-type configuration, such as 21(OH)pD, was shown to inhibit the growth of the SK-MEL-188b line. It has to be underlined that this analog, also showed high potency against two other melanoma cell lines in the study (A375 and WM98) and minimal stimulation of CYP24A1. This indicates that the antiproliferative properties of 21-OHpD are independent on its action on the VDR, which is consistent with our previous findings on pregnacalciferol derivatives acting as poor activators of VDR per VDR translocation studies and molecular modeling [69]. One of the possible mechanisms of 21(OH)pD action could include an activation of non-genomic pathways [25,80], including those linked to the PDIA3 protein [81,82]. However, interaction with other nuclear steroid receptors cannot be excluded. In addition, other receptors such as retinoic orphan acid receptors (ROR)α and γ can be considered since vitamin D hydroxyderivatives can act as reverse agonists on these receptors [62,83] and related pregnalumisterol derivatives can also act on RORα and RORγ [15]. These considerations require future in-depth investigations to define specific receptors for pD compounds (secosteroids with shortened side-chain).

Two other melanoma lines were responsive to vitamin D (WM98 and A375) and showed relatively high expression levels of *VDR* transcript. Thus, the studies presented above on three melanoma lines are consistent with reports showing that the expression of *VDR* is the key factor responsible for the antitumor activities of 1,25(OH)_2_D_3_ [18,47,84,85]. Of note is that its decreased expression correlates with advanced melanoma staging, progression and decreased overall patient survival and disease-free survival time [45,46]. Accordingly, *VDR* polymorphism [41,42,72] and decreased levels of 25(OH)D_3_ in the serum [33,86] positively correlate with melanoma prevalence and poor prognosis.

Here, we show that sensitivity to tested vitamin D_3_ hydroxyderivatives with full side-chains depends on *VDR* expression and its alternative splicing. Interestingly, sensitive melanoma lines (A375, WM98) expressed all three major *VDR* splicing variants, suggesting that they may play an important but not necessarily identical function in vitamin D_3_ signaling. Further studies are required to elucidate the significance of the expression of *VDR* splicing variants. Furthermore, the expression of vitamin D_3_-related genes was altered by vitamin D_3_ analogs, and the effects on the expression of *VDR, RXR, PDIA3, CYP2R1* or *CYP24A1* were stronger in WM98 melanoma cells in comparison to the A375 line. This phenomenon could explain higher sensitivity of WM98 to 1,25(OH)_2_D_3_ in comparison to A375 line.

In summary, low calcemic vitamin D_3_ analogs such as 25(OH)D_3_, 21(OH)pD or calcipotriol showed similar antiproliferative activity to 1,25(OH)_2_D_3_ in melanoma cell lines expressing VDR spliced variants. Furthermore, the short side-chain analog 21(OH)pD was found to be superior among vitamin D analogs and was the only one, which inhibited the growth of the melanoma subline negative for VDR, indicating a mechanism of action that is VDR-independent.

## 4. Materials and Methods

### 4.1. Cell Lines and Vitamin D Analogs

In our study, we determined the inhibitory effects of vitamin D_3_ analogs against immortalized human melanoma cell lines SK-MEL-188b, WM98 and A375. SK-MEL-188b cells were cultured in F10 medium supplemented with 10% fetal bovine serum (FBS, Sigma, Poznan, Poland) and 1× antibiotic-antimycotic solution (Anti-Anti, Sigma). A375 and WM98 cell lines were cultured in Dulbecco's Modified Eagle Medium (DMEM) supplemented with 10% FBS and 1% anti-anti. To eliminate the influence of sterols present in fetal bovine serum in the experiments, we used 5% charcoal-stripped FBS (GE Healthcare Life Sciences, Warsaw, Poland). Vitamin D_3_ analogs 1,25(OH)_2_D_3_, 25(OH)D_3_ and calcipotriol were acquired from the Pharmaceutical Research Institute (Warsaw, Poland), while the new short side analog 21(OH)pD was synthesized in collaboration with ProChimia Surfaces (Gdynia, Poland) as described previously [49,50]. The chemical structures of these secosteroids are presented in Figure 1.

### 4.2. SRB Assay

Melanoma cells were seeded on 96-well plates at a density of 8000 per well in an appropriate medium supplemented with 5% charcoal-stripped FBS and 1× anti-anti solution. After 24 h, the medium was replaced with the fresh one containing vehicle and serial dilutions of vitamin D_3_ analogs at concentrations from 10 pM to 1 μM. Following incubation for 48 h, 100 μL of 20% TCA (trichloroacetic acid) was added and cells were incubated for 1 h in 4 °C. The medium was discarded and cells were washed 5 times with sterile water. Afterwards, cells were dried and 100 μL of SRB (0.4 g sulforhodamin B in 100 mL 1% acetic acid) was added into the plate wells for 15 min incubation at room temperature. Next, cells were washed 5 times with 1% acetic acid, dried and resolved with 150 μL of 10 mM Tris (pH = 10.5) for 10 min in room temperature. The absorbance was measured spectroscopically at 570 nm with 96-well plate reader (BioTek, Winooski, VT, USA).

### 4.3. Classical PCR and Real-Time PCR Analysis

SK-MEL-188b, A375 and WM98 melanoma cells were treated with 1,25(OH)_2_D_3_, 25(OH)D_3_ and calcipotriol (only WM98 line) for 24 h (Figure 5 and Figure S1) or collected without treatment (Figure 3 and Figure 4). RNA was isolated using a Total RNA Kit (A&A Biotechnology, Gdynia, Poland). Reverse transcription (500 ng RNA/reaction) was carried out with a RevertAid™ First Strand cDNA Synthesis Kit (Fermentas, Vilnius, Lithuania). Classic PCR and real-time PCR were performed using 5-fold diluted cDNA and 2× PCR Master Mix (A&A Biotechnology) or real time 2× PCR Master Mix SYBR Set A, B (A&A Biotechnology). The primers to amplify fragments of *ACTB*, *VDR*, *RXR*, *PDIA3*, *CYP27A1*, *CYP2R1*, *CYP3A4*, *CYP27B1*, *CYP24A1*, *CYP24SV* genes were designed with Primer Quest software (Integrated Device Technology, San Jose, CA, USA) (Table S1). The data was collected on a MJ Mini BioRad cycler (BioRad, Hercules, CA, USA) or Termocycler StepOne Real-Time PCR Systems (Life Technologies, Carlsbad, CA, USA). PCR products were visualized using the Mupid-One electrophoresis system (BioRad) and ethidium bromide staining.

### 4.4. VDR Translocation

The melanoma A375 cell line was used to study the VDR receptor translocation to nucleus after vitamin D_3_ analog treatment. Cells were transduced by pLenti-CMV-VDR-EGFP-pgkpuro plasmid, where VDR and GFP were expressed as a fusion protein in cytoplasmic compartment. Cells were cultured on 12 wells plate (0.5 × 10^5^ cells/well) in DMEM supplemented with 5% charcoal-stripped FBS and 24 h later medium was replaced with DMEM containing 1,25(OH)_2_D_3_, calcipotriol and 21(OH)pD at 0.01 µM, 0.1 µM or 1 µM concentrations. After 1 h of incubation VDR translocation from cytoplasm to nucleus was determined with fluorescent Eclipse TE300 microscope (Nikon, Tokyo, Japan).

### 4.5. Statistical Analysis

SRB viability data was presented using one-way Anova test to compare experimental groups: cells were treated with vitamin D_3_ analogs with a concentration from 0.01 nM to 1000 nM with control samples (GraphPad Software). The antiproliferative potency of vitamin D_3_ analogs were compared by the calculation of EC50 for every compound (half maximal effective concentration). Real-time PCR data was analyzed with the comparative ΔΔ-Ct method normalized to the reference gene *ACTB*. As a control, a probe with sterile water was used instead of cDNA. Data are presented as mean ± SD (*n* = 4–6). Student’s *t*-test (for two groups) or one-way ANOVA with appropriate post-hoc test (for more than two groups) were used to analyze data using Excel (Microsoft) or Prism 7.00 (GraphPad Software), respectively. Statistically significant differences are denoted with asterisks: *p* < 0.05 (* *p* < 0.05; ** *p* < 0.01; *** *p* < 0.0001).

## Supplementary Materials

Supplementary materials can be found at http://www.mdpi.com/1422-0067/19/9/2583/s1.

## Abbreviations

1α,25(OH)_2_D_3_	1α,25-dihydroxyvitamin D_3_ (calcitriol)
21(OH)pD	21-hydroxypregnacalciferol
25(OH)D_3_	25-hydroxyvitamin D_3_ (calcifediol)
7-DHC	7-dehydrocholesterol (provitamin D_3_, cholesta-5,7-dien-3β-ol)
7-DHP	7-dehydropregnenolone
MARRS receptor	Membrane-Associated Rapid Response to Steroid binding protein (other names: ERp57, GRp58, Pdia3)
PDIA3	Protein disulfide-isomerase A3
ROS	reactive oxygen species
UVA/B	ultraviolet radiation A and B
VDR	vitamin D receptor
VDRE	vitamin D response elements
