# Supplementary material for: Antiproliferative Activity of Non-Calcemic Vitamin D Analogs on Human Melanoma Lines in Relation to VDR and PDIA3 Receptors
Source: Int J Mol Sci. 2018 Aug 31;19(9):2583. doi: 10.3390/ijms19092583 (PMC6163194; doi:10.3390/ijms19092583)
Supplement: Supplementary file 1 [file ijms-19-02583-s001.pdf]

Supplementary Materials

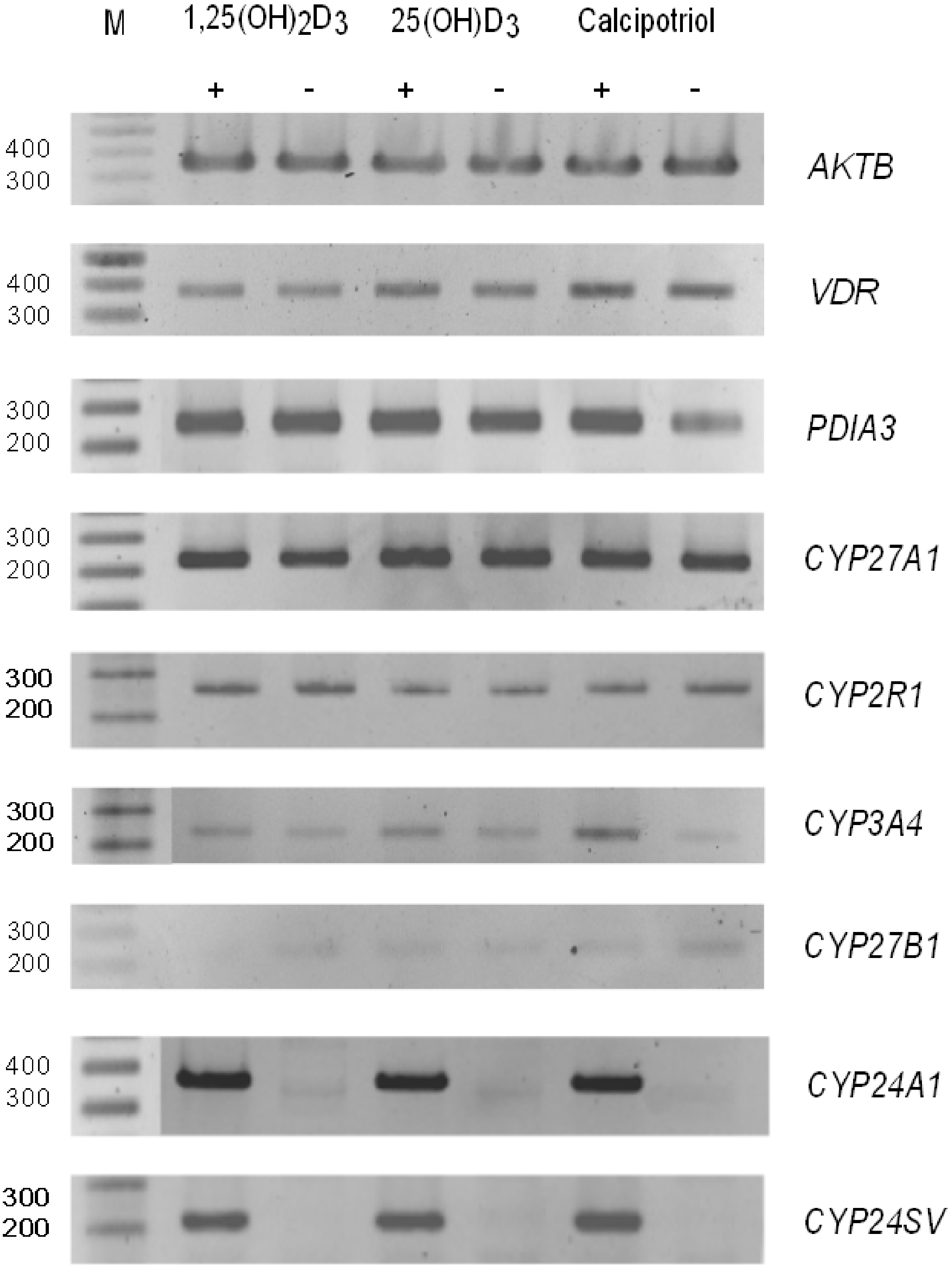

Figure S1. Analysis of induction of *VDR*, *PDIA3*, *CYP27A1*, *CYP2R1*, *CYP3A4*, *CYP27B1*, *CYP24A1* and *CYP24SV* (mRNA) after 1,25(OH)$_2$D$_3$, 25(OH)D$_3$ or calcipotriol treatment (1 μM, 24 h) using WM98 cell line. The relative amount of mRNA was determined by semiquantitative PCR method normalized to AKTB mRNA level. The specificity of the PCR products were evaluated in relation to molecular weight marker (M) to confirm the lengths of amplicons as presented in Table S1.

Table S1. Sequences of design primers used to amplify fragments of genes (from 180 base pairs to 384 base pairs) related with vitamin D response. Primers were designed using human melanoma genes sequences from NCBI BLAST, http://blast.ncbi.nlm.nih.gov/.Melting temperature of primers calculated using Primer Quest Software. GAPDH primers were supplemented to RevertAid™ First Strand cDNA Synthesis Kit (Fermentas. Vilnius, Lithuania).

	Sequence	Name	Organism	Temp. (°C)	b.p.
1	GCTCGTCGTCGACAACGGCTC	*AKTB F*	Homo sapiens	60,2	353
	CAAACATGATCTGGGTCATCTTCT	*AKTB R*	Homo sapiens	54	
2	CAAGGTCATCCATGACAACTTTG	*GAPDH F*	Homo sapiens		
	GTCCACCACCCTGTTGCTGTAG	*GAPDH R*	Homo sapiens		
3	CCAGTTCGTGTGAATGATGG	*VDR Pr 5 F*	Homo sapiens	59,96	384
	GTCGTCCATGGTGAAGGA	*VDR Pr 5 R*	Homo sapiens	57,83	
4	TGAACTTGCATGAGGAGGAGCA	*VDR Pr 1 F*	Homo sapiens	58.4	132
	TGTACGTCTGCAGTGTGTTGGA	*VDR Pr 1 R*	Homo sapiens	58.6	
5	GCCACTGGCTTTCACTTCAATGCT	*VDR Pr 2 F*	Homo sapiens	60,1	180
	TCATCATGCCGATGTCCACACA	*VDR Pr 2 R*	Homo sapiens	58,6	
6	TGTCGATGGTGCTCAGAACTGCT	*VDR Pr 3 F*	Homo sapiens	60,3	386
	TCATCATGCCGATGTCCACACA	*VDR Pr 3 R*	Homo sapiens	58,6	
7	AACAGCTTGTCCACCCGCC	*VDR Pr 4 F*	Homo sapiens	60,7	532-b*
	TCATCATGCCGATGTCCACACA	*VDR Pr 4 R*	Homo sapiens	58,6	410-c*
8	TCAGGAAACATGGCTTCCTTCACC	*RXRα F*	Homo sapiens	71	175
	ACAAGGACTGCCTGATTGACAAGC	*RXRα R*	Homo sapiens	69,4	
9	CTCCGACGTGCTAGAACTCA	*Pdia3 F*	Homo sapiens	63,2	204
	CAGGTGTTAGTGTTGGCAGT	*Pdia3 R*	Homo sapiens	60,3	
10	GGCAAGTACCCAGTACGG	*CYP27A1 F*	Homo sapiens	56,52	292
	AGCAAATAGCTTCCAAGG	*CYP27A1 R*	Homo sapiens	53,07	
11	AGAGACCCAGAAGTGTTCCAT	*CYP2R1 F*	Homo sapiens	61,6	259
	GTCTTTCAGCACAGATGAGGTA	*CYP2R1 R*	Homo sapiens	60,9	
12	AAGGCACCACCCACCTATGATACT	*CYP3A4 F*	Homo sapiens	67,3	197
	TACTTTGGGTCACGGTGAAGAGCA	*CYP3A4 R*	Homo sapiens	70,5	
13	TGTTTGCATTTGCTCAGA	*CYP27B1 F*	Homo sapiens	54,63	227
	CCGGGAGAGCTCATACAG	*CYP27B1 R*	Homo sapiens	56,79	
14	GCAGCCTAGTGCAGATTT	*CYP24A1 F*	Homo sapiens	53,97	335
	ATTCACCCAGAACTGTTG	*CYP24A1 R*	Homo sapiens	51,3	
15	TCCTGAAGTTGCAGCTGGAGT	*CYP24SV F*	Homo sapiens	66,3	213
	GAGCTCATCTATTCTGCCCATA	*CYP24SV R*	Homo sapiens	62,1	

* Depends on VDR isoform PCR product consist of 410 or 532 base pairs.
